# Bat Motion can be Described by Leap Frogging

**DOI:** 10.1007/s11538-023-01233-5

**Published:** 2024-01-10

**Authors:** Lucy Henley, Owen Jones, Fiona Mathews, Thomas E. Woolley

**Affiliations:** 1 Cardiff School of Mathematics Cardiff University, Senghennydd Road, Cardiff, CF24 4AG UK; 2 University of Sussex, John Maynard Smith Building, Falmer, Brighton, BN1 9RH UK

**Keywords:** Bat motion, Partial differential equations

## Abstract

We present models of bat motion derived from radio-tracking data collected over 14 nights. The data presents an initial dispersal period and a return to roost period. Although a simple diffusion model fits the initial dispersal motion we show that simple convection cannot provide a description of the bats returning to their roost. By extending our model to include non-autonomous parameters, or a leap frogging form of motion, where bats on the exterior move back first, we find we are able to accurately capture the bat’s motion. We discuss ways of distinguishing between the two movement descriptions and, finally, consider how the different motion descriptions would impact a bat’s hunting strategy.

## Introduction

Bats are an important part of ecosystems around the world, playing a vital role in controlling insect populations, seed dispersal and pollination (Kunz et al. 2011). There are more than 1270 bat species worldwide, occupying a huge range of habitats on each continent except Antarctica and bats are among the most ecologically diverse groups of mammals on the planet (Kunz and Fenton 2005).

Insectivorous bat species, such as the 18 species found in the UK, feed primarily on airborne insects and contribute to suppressing insect populations including agricultural pests and species, such as mosquitoes, which can spread diseases. Due to their role as predators, they are sensitive to changes in the population of insects and can therefore act as ecological indicators of biodiversity and pollution (Jones et al. 2009).

Bat activities and populations are sensitive to a number of human driven factors such as light and noise pollution and climate change. Habitat fragmentation due to roads and building work can also reduce foraging opportunities and lead to a significant risk of population decline (Rossiter et al. 2000).

In the maternity season during summer, large numbers of bats tend to group together in a single roost to have young, and as such it is imperative that these roosts are identified and protected. The disturbance of bat roosts has been identified as a significant cause of the population decline of bat species in Europe during the past century (Stebbings 1988; Hutson and Mickleburgh 2001). As a result, bats are protected by law in Europe under the EUROBATS agreement (Marnella and Presetnik 2010) and under domestic law in the UK (Wildlife and Countryside Act 1981; Conservation of Habitats and Species Regulations 2017).

We seek to aid bat conservation through understanding bat movement. If we understand how bats travel away from and back to their roosts we can then use this to predict roost locations (Henley 2022). We will focus on using deterministic models (Voigt et al. 2017a; Woolley et al. 2012; Woolley 2011) to describe the movement of greater horseshoe bats, a species that is classified as Near Threatened across Europe owing to significant declines in its distribution and abundance over the last 50 years (Jones et al. 2009). Great Britain, and particularly south-west England are a stronghold for the species, and several detailed ecological studies have been conducted in this region (Finch et al. 2020a; Mathews 2009; Froidevaux et al. 2017). Greater horseshoe bats roost predominantly in caves. However, particularly in the northern part of their distribution, they also frequently form maternity colonies in the loft spaces of barns, stable blocks and large houses.

**Fig. 1:**
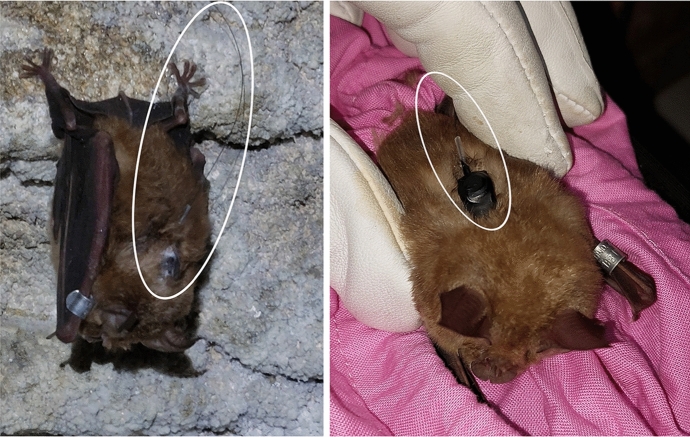
Greater horseshoe bats with radio transmitters glued to their backs. The radio transmitters have very thin antennae, and are highlighted in white. Photographs taken by Professor Fiona Mathews

Various methods are employed for identifying, or locating bat roosts, and in general, they all entail significant labour. Radio-tracking surveys are a common approach for studying bat movement, habitat preferences, and to track animals back to their roosts (Bontadina et al. 2002; Encarnação et al. 2005; Kunz and Parsons 1988). This approach entails humanely capturing bats in flight, using mist nets or harp traps and attaching a small radio transmitter to the bat’s back typically with surgical glue. The transmitter must be less than 5% of the bat’s weight in order to avoid disrupting flight patterns (Brigham 1988). Images of a typical transmitter attached to a greater horseshoe bat are shown in Fig. 1.

Upon releasing a bat, the signal from their transmitter is picked up by field workers using scanning radio receivers. Triangulation is generally used to increase the accuracy of the bat’s position estimate, which requires a minimum of two field workers to simultaneously record a bearing. However, with bats reaching flight speeds exceeding 20mph ($\approx$ 9 m/s) (Jones 1995), recording simultaneous bearings by multiple workers is often problematic, introducing potential human error. Field workers shadow the bat, attempting to maintain contact throughout the night, continuously scanning for the signal and recording location data until either the signal is lost or the bats return to their roost. Notably, bat locations are recorded at irregular intervals, occurring only when the signal is detected.

Due to transmitter range limitations, workers must remain in close proximity to the bat, typically within 1–2 km, or even closer if the bat roosts within a building or underground structure. This can be particularly challenging in rural environments with obstacles like impassable waterways, hedgerows and hills. Furthermore, transmitters have limited battery life and are often lost before the survey concludes, potentially truncating data collection (O’Mara et al. 2014). Additionally, given the numerous potential roost locations, conducting physical surveys of each site is not always feasible.

Mathematical models are an invaluable tool in understanding ecology as they help us to derive the mechanisms that lead to certain patterns of behaviour (Ovaskainen et al. 2016). There are many possible formalisms, such as stochastic or deterministic, continuous or discrete, depending on the population and behaviour (Murray 2003; Collins-Hooper et al. 2012; Woolley et al. 2013). Partial differential equation (PDE) models can provide a useful approximation to real life, whilst simplifying the mathematics by excluding noise (Belmonte-Beitia et al. 2013). However, as there are a finite number of animals in a population, adding stochastic dynamics can sometimes improve the model approximations (Woolley et al. 2011a, b; Hill et al. 2021).

In this paper, we focus on diffusion-type models for bat movement. Diffusion-type models have been used for decades to describe animal movement, and can be formalised with stochastic processes, or with PDEs (Patterson et al. 2017; Ovaskainen et al. 2016; Woolley et al. 2011c; Woolley 2011). With improvements in electronic animal tracking devices in recent years, increasingly detailed data can now be gathered and used to inform complex models of animal movement.

Stochastic processes are able to effectively describe movement of individual animals under the influence of group dynamics and landscape effects (Patterson et al. 2017; Woolley et al. 2014, 2022). The Ornstein-Uhlenbeck (OU) process was first introduced in 1930 as an adaptation of Brownian motion which includes an overall drift towards a specific location (Uhlenbeck and Ornstein 1930). The OU process is a stochastic process with two components, a random diffusion element and a deterministic convection or drift element. The first method for modelling animal locations using a stochastic process in continuous time in 1977 Dunn and Gipson (1977) was derived from the OU process, and provided a description of an animal’s home range, defined as the smallest geographical area in which the animal spends a fixed proportion of time (Jennrich and Turner 1969). Since 1977, stochastic processes have been used to study movement on various scales ranging from the microscopic movement of cells (Woolley et al. 2014), migration of large land mammals such as elk (Preisler et al. 2004) to constructing flight models of bumblebees (Lenz et al. 2013).

However, following the dynamics of each individual in a colony can prove to be computationally expensive, particularly when studying large populations under the influence of multiple external factors (Patterson et al. 2017). In contrast, PDE models, also frequently used to model a wide range of biological systems, reduce complexity by excluding individual dynamics, focusing only on the dynamics of the group as a whole (Murray 2003; Woolley 2017). In the context of animal movement, PDEs have been applied to a range of problems including home-range formation and territory use (Potts and Lewis 2014), insect dispersal (Ovaskainen et al. 2008) and flocking behaviour (Eftimie et al. 2007). PDE diffusion models have been used to model the movement of bats in both homogeneous and heterogeneous landscapes (Cvikel et al. 2015; Kerches-Rogeri et al. 2021). Whilst PDEs are highly useful in analysing the properties of ecological systems, their use in empirical and statistical ecology is much less common (Potts and Schlägel 2020). This is likely due to the difficulties in choosing PDE models and fitting them to data. Fitting parameters to a PDE model often requires numerically solving the PDE for various parameter values (Ferguson et al. 2016), and as a result simpler or easier to solve stochastic process models are often favoured by non-mathematical ecologists.

Overall, both stochastic process and PDE models are useful for solving different problems: stochastic models can effectively describe individual dynamics, and can be simpler to simulate by ecologists, whilst PDE models exclude individual variation and can provide useful approximations.

Although our mathematical approaches are fairly standard, to our knowledge, it is the first time that these techniques have been applied to bat motion. Moreover, the motion characteristics of the mean squared displacement during the returning phase are unusual and require additional temporally varying factors, such as shrinking domain models that have previously not been applied in this manner. In this paper we use time-location data from radio-tracking studies that track bat motion from when they first leave their roost at sunset to when they return in the morning. By extracting the mean squared displacement (the ensemble average of squared displacement from the roost over time) from the data we see two distinct movement phases, an initial linear dispersal followed by a gradual return to the roost. We use this data to develop PDE models to describe motion for each movement phase. Diffusion models are discussed to describe the dispersal of bats away from the roosts. Convection-diffusion models are widely used in ecology to model population migration, however we will show here that a temporally homogeneous convection-diffusion model is not consistent with radio-tracking data, whilst an non-autonomous convection-diffusion model can be used to match the data. However, due to inconsistencies with bat behaviour narrative we develop a shrinking domain diffusion model (Crampin et al. 2002a; Crampin 2000; Crampin and Maini 2001a, b; Crampin et al. 1999) that provides a better description of bat movement during their returning phase.

## Radio-Tracking Survey

A radio-tracking study was conducted at three greater horseshoe bat roosts in Devon to study the usage of land surrounding the roosts (Mathews 2009). Twelve bats were fitted with radio tags and studied over 24 nights during May and June 2016 by a team of trained volunteers and ecologists, such that between 2 and 6 people were tracking on each night. Due to a limited number of workers and limited battery life on the tags, each bat was not tracked every night. Instead, effort focused on one to two bats at one time, attempting to maintain close contact. Four day roosts were used by bats in the study, with some bats using different roosts on different days. The roost used by each bat was not identified on every night.

For this analysis, only data from nights when a bat’s beginning and ending roost were the same is used. As such, only the trajectories of 7 bats over 14 nights were used consisting of a total of 25 individual trajectories containing a total of 328 recordings. All of the data and accompanying MATLAB codes to produce the figures can be found at https://github.com/ThomasEWoolley/Bat_motion.

**Fig. 2:**
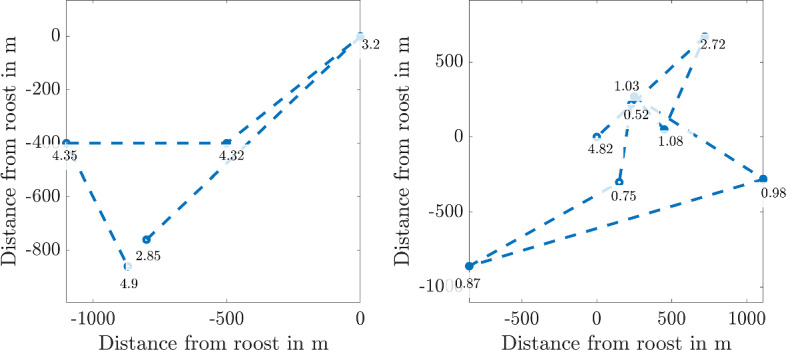
The locations of the same bat over two nights during the survey. The roost has been normalised to be at the origin in each case. The circles represent detections and the numbers next to them represent the time in hours after sunset that the bat was detected at the given location

As an example of the data we illustrate the trajectory of a bat over two different nights in Fig. 2. We observe that the bat visits different areas whilst foraging, taking a different route on each night. Further, we note that the number and time of recordings varies each night, which is one of the difficulties of bat detection.

**Fig. 3:**
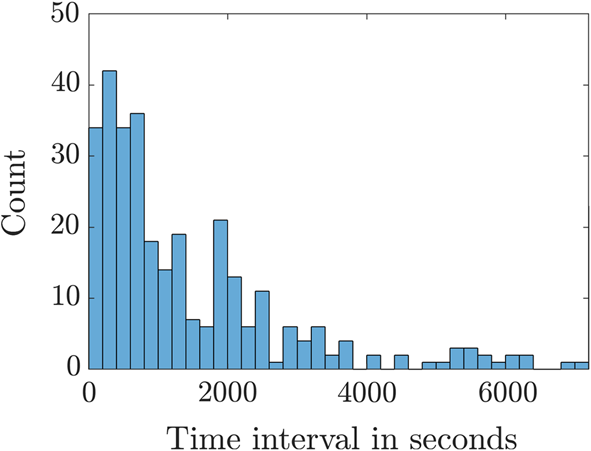
A histogram of the time intervals between consecutive recordings

A histogram of time intervals between consecutive recordings is shown in Fig. 3, demonstrating the irregularity of recording intervals. We will use the mean-squared distance (MSD) from the roost as a function of time to summarise the motion features and thus fit the movement models to the data. In order to calculate the MSD at a given time, we require regularly spaced recordings. The locations were linearly interpolated between recordings at intervals of $\Delta t = 200$ seconds, as the distribution in Fig. 3 peaks between 0 and 400 s.

**Fig. 4:**
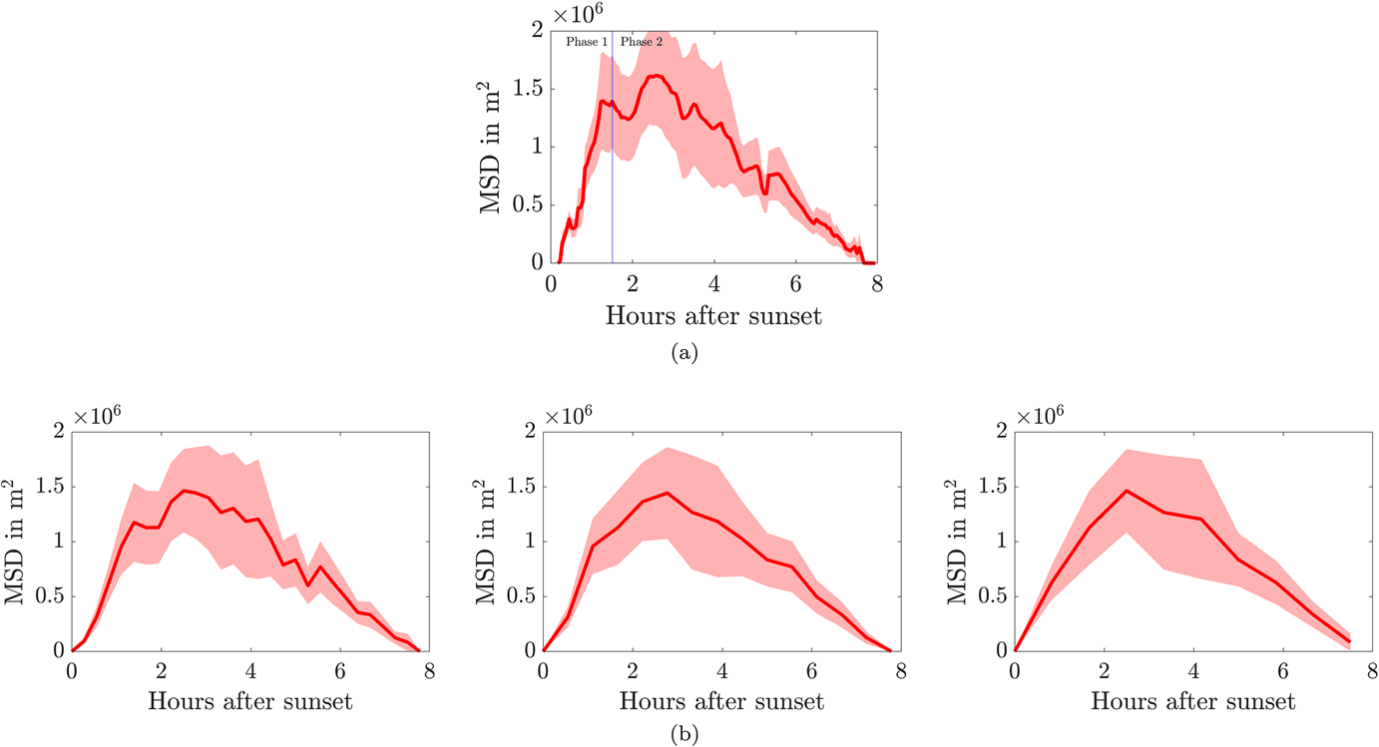
**a** The mean-squared distance (MSD) for all radio tracked bats interpolated at $\Delta t=200$s. **b** The MSD for all radio tracked bats interpolated at $\Delta t=1000$, 2000 and 3000 s, from left to right, respectively. The red line is the MSD trajectory and the ribbon represents the mean±standard error of the squared displacement trajectory data

The MSD from the roost was calculated from the interpolated positions using

1 $$\begin{aligned} \langle r^2(t)\rangle = \frac{1}{N(t)} \sum _{i=1}^{N(t)} |\varvec{x_i}(t)-\varvec{x_i}(0)|^2, \end{aligned}$$

where $\varvec{x_i}(t)$ is the location (*x*, *y*) of bat *i* at time *t* and *N*(*t*) is the number of trajectories that exist at time *t*. Due to the trajectories being chosen such that the bats are recorded as having started and ended at the same roost the MSD (shown in Fig. 4a) begins and ends at zero. Another statistic we will be frequently using to provide confidence intervals about the MSD is the Standard Error, $\sigma _{SE}$, which is the standard deviation of the squared displacement of individual bat tracks, $\sigma$, divided by the square root of the number of tracks, *N*(*t*), thus, $\sigma _{SE}=\sigma /\sqrt{N(t)}$.

In much of the following we will be using an interpolation interval of $\Delta t=200$s. We note that increasing $\Delta t$ (shown in Fig. 4b) leads to a reduction in used data, however, it does not change the overall shape of the curve from Fig. 4a.

The data in Fig. 4a indicates two movement phases, an initial rapid dispersal from the roost, followed by a gradual return whilst bats are foraging. We will separate these two phases at a fixed transition point of $t_s=1.5$ hours, which matches the ecologists rule of thumb that the first 90 min after sunset is the major foraging period for greater horseshoe bats, thus, it is the time we would expect most dispersion. At the end of Sect. 3.2.3 we will revisit this assumption and additionally fit this transition time.

During phase 1, for $0 \le t \le 1.5=t_s$ hrs, the MSD seems to increase linearly as bats are dispersing. The standard error grows during this phase as the bats spread out. During phase 2, for $t_s=1.5< t < 8$ hrs, the MSD decreases as bats move back towards the roost, shrinking to zero at $t \approx 8$ hrs. The variation also shrinks to zero during this phase as bats start to converge on the roost.

Note we will frequently describe the shape of the phase 2 MSD as convex, meaning that, on average, it does not initially tend to $MSD=0$, but rather it can either be seen to stay constant, or perhaps even increase, before decreasing to zero. We will compare this generic shape with the MSDs from convection-diffusion equations with fixed parameters in Sect. 3.2, which decrease rapidly to $MSD=0$, which will be known as concave shapes. Our task will be to develop the convection-diffusion framework in a couple of directions to reproduce the convex MSD shape.

In the next section various models for each phase will be compared. Although the data is recorded at irregular time points and stochastic in nature, we will assume that the underlying probability distribution of the ensemble dynamics is continuous in both space and time. Therefore, we will be able to use partial differential equations to analytically describe the evolution of the probability distribution.

## Modelling Methods

### Phase 1: Dispersal

Diffusion models describe agents as random walkers and are widely used to model dispersal animal movement for a number of species (Ovaskainen et al. 2016). In this case, we wish to describe the dispersal during phase 1 of movement as bats fly away from the roost to the surrounding areas. It is commonly accepted that bats tend to remain within an area around the roost known as the Core Sustenance Zone, CSZ, and will forage within this area for the majority of the night (Trust 2016). As a result, a diffusion model on a bounded domain is considered here.

#### Diffusion in Polar Coordinates

Although bats are not restricted to a single movement plane, as they are able to fly in any direction in 3D, we will not include the third dimension here, as height is not measured in the radio-tracking survey. As the bats are unrestricted in two dimensions we will assume their motion is unbiased in terms of direction leading us to use a diffusion model in polar coordinates.

If the roost is at $(x_0,y_0)$ and bats leave the roost at time $t =0$, the 2D diffusion equation describes the probability density $\phi (x,y,t)$ of finding a bat at position (*x*, *y*) at time *t*. Explicitly

2 $$\begin{aligned} \frac{\partial \phi (x,y,t)}{\partial t} = D \nabla ^2 \phi (x,y,t), \end{aligned}$$

where $\nabla ^2$ is the Laplacian, *D* is the diffusion coefficient, a positive constant that quantifies the rate of spread. The CSZ is denoted by $\Omega \subset {\mathbb {R}}^2$ and modelled as a disk of radius *R* centred around the roost. Since the domain and initial condition are angularly symmetric, $\phi$ is only dependent on *r* and not on the angle, and we drop the angular dependence from the Laplacian. The diffusion equation in radial polar coordinates is then given by

3 $$\begin{aligned} \frac{\partial \phi (r,t)}{\partial t} = \frac{D}{r} \frac{\partial }{\partial r} \left( r \frac{\partial \phi (r,t)}{\partial r} \right) , \end{aligned}$$

where *r* is the distance from the roost, given by $r=\sqrt{(x-x_0)^2 + (y-y_0)^2}$. The initial condition,

4 $$\begin{aligned} \phi (r, t = 0) = \delta _0(r), \end{aligned}$$

specifies that all bats begin the night at the roost, at position $r=0$. The boundary condition,

5 $$\begin{aligned} \frac{\partial \phi (r=R,t)}{\partial r} = 0, \end{aligned}$$

specifies zero-flux across the boundary such that bats cannot enter or leave the boundary. Of course, in reality there is no physical boundary, *R* simply represents some maximum distance that the bats tend not to violate.

Equation (3) cannot be solved for all space and time under the given initial and boundary conditions. However, we can extract information regarding how displacement averages evolve over time.

#### Mean Squared Displacement in the Diffusion Model in Polar Coordinates

Given a probability distribution, $\phi$, the moments of $\phi$ are defined by (Curtiss 1942)

6 $$\begin{aligned} \langle r^n\rangle = \int _{\Omega }r^n \phi (r,t) d\omega . \end{aligned}$$

where $\langle . \rangle$ denotes that a mean value is being taken as in Eq. (1). Thus, even though we are unable to calculate $\phi$ exactly, we are able to make some analytical headway in understanding the MSD through calculating the second moment (Anh et al. 2009; Yun 2018; Grebenkov 2019). Taking the time derivative of both sides, we can then use Eq. (3) to provide the following derivation

7 $$\begin{aligned} \frac{d}{dt} \langle r^2\rangle&= \int _0^{2\pi } \int _0^{R} r^3 \frac{\partial \phi }{\partial t} dr d\theta ,\nonumber \\&= \int _0^{2\pi } \left( \underbrace{\left[ D r^2 \left( r \frac{\partial \phi }{\partial r}\right) \right] _0^{R}}_{=0} - \int _0^{R} 2rD \left( r \frac{\partial \phi }{\partial r} \right) dr \right) d\theta , \nonumber \\&= \int _0^{2\pi } \left( \left[ -2r^2D \phi \right] _0^{R} + \int _0^{R} 4rD \phi dr \right) d\theta , \nonumber \\&= 4D( 1- \pi R^2 \phi (R,t)) . \end{aligned}$$

Finally, integrating with respect to time gives

8 $$\begin{aligned} \langle r^2\rangle = 4D \left( t - \pi R^2 \int _0^t \phi (R,\tau ) d \tau \right) . \end{aligned}$$

Depending on the sizes of *R* and *D* then for *t* small, $\phi (R,t) \approx 0$, since the probability of reaching the boundary over a short period of time is small due to the compact initial condition. Therefore, while

9 $$\begin{aligned} t \ll \frac{R^2}{D}, \end{aligned}$$

the expected MSD for diffusion is approximately equal to the first term of Eq. (8), meaning that the MSD is approximately proportional to time,

10 $$\begin{aligned} \langle r^2\rangle \approx 4Dt. \end{aligned}$$

As noted in Sect. 2 the MSD in Fig. 4a is indeed linear during phase 1, and therefore consistent with a diffusion model.

An expression for mean-squared displacement over a long time scale can also be derived using Eq. (6). Over long time scales, we expect the probability density to be uniformly spread across the domain,

11 $$\begin{aligned} \phi (r,t) = \frac{1}{\pi R^2}. \end{aligned}$$

Substituting this into Eq. (6) gives

12 $$\begin{aligned} \langle r^2\rangle&= \frac{1}{\pi R^2}\int _{0}^{2\pi } \int _{0}^{R} r^3 dr d\theta = \frac{1}{2} R^2, \end{aligned}$$

and therefore the mean squared displacement is constant over long time scales.

**Fig. 5:**
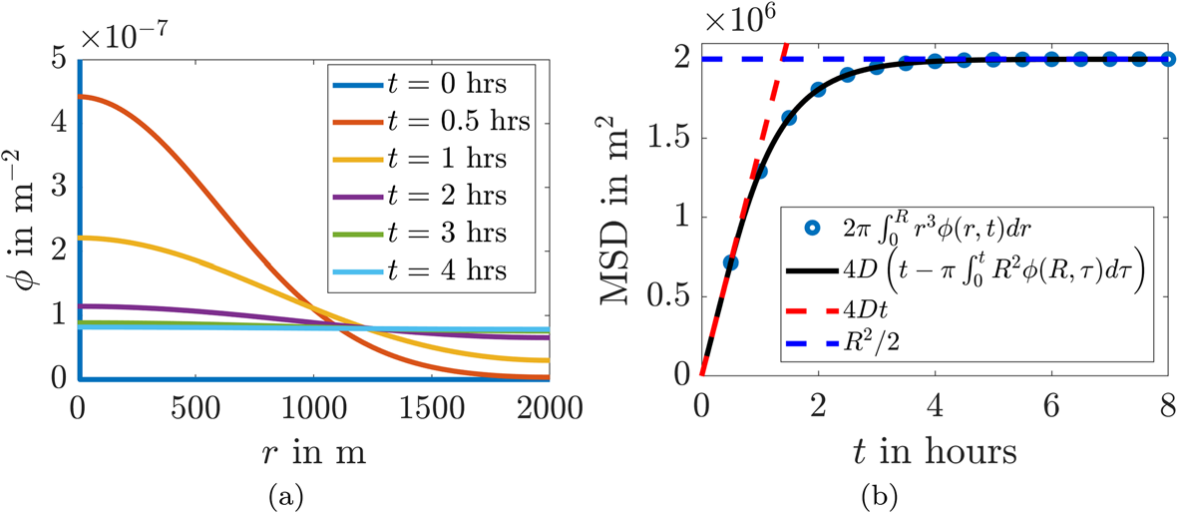
**a** Simulation of Eq. (2), the diffusion in polar coordinates, and **b** derived MSD from Eqs. (6) and (8) compared with approximations from Eqs. (10) and (12). Parameter values are $D=100$ m^2^/s and $R = 2000$ m (Color Figure Online)

To illustrate the accuracy of these approximations, we simulate Eq. (3) using pdepe from MATLAB R2022b (Inc. 2022; Coleman 2013; Stanoyevitch 2005) and extract the MSD directly. Figure 5a illustrates the radial profiles of the probability density $\phi$ over a number of times shown in the legend. We can observe that the initial condition approximates a delta function as specified by Eq. (4) the evolving shape smoothly transports density from local peaks to local troughs, eventually tending to a uniform spread of density across the domain. Figure 5b illustrates the excellent comparison between the methods of calculating the MSD through Eqs. (6) and (8), as well as the short time and long time approximations, namely the MSD grows linearly before plateauing to a constant value, consistent with Eqs. (10) and (12).

### Phase 2: Return to Roost

The diffusion model described in Sect. 3.1 explains the initial dispersal in phase 1, however it cannot explain the decrease in MSD for phase 2 (see Fig. 4a). For the second phase of movement we consider adding a convection term to the diffusion equation, which describes a drift in a particular direction, or towards a specific location. These models are commonly used to describe animal movement in response to external factors, for example a drift towards patches of high resources, or away from predators (Ovaskainen et al. 2016).

We will demonstrate that a convection-diffusion model with fixed parameters is unable to reproduce the observed MSD curve in Fig. 4, but allowing the diffusion and convection terms to depend on time does allow the equation to generate a good fit. As an alternative hypothesis we will show that a diffusion model in a shrinking domain provides an equally good fit to the data. Thus we look to the data to see if there is a way of separating the mechanisms.

#### A Convection–Diffusion Model in Two Dimensions

During phase 2 of movement, the MSD is decreasing as bats return towards their roost location $(x_0,y_0)$ at $r=0$. We will first consider a convection-diffusion model with fixed parameters to describe this drift. The convection-diffusion equation is given by (Murray 2003)

13 $$\begin{aligned} \frac{\partial \phi }{\partial t} = D \nabla ^2\phi + \nabla \cdot (\varvec{v} \phi ), \end{aligned}$$

where $\nabla$ is the gradient and $\varvec{v}$ is the vector flow velocity. The initial and boundary conditions are once again a delta function and zero-flux.

**Fig. 6:**
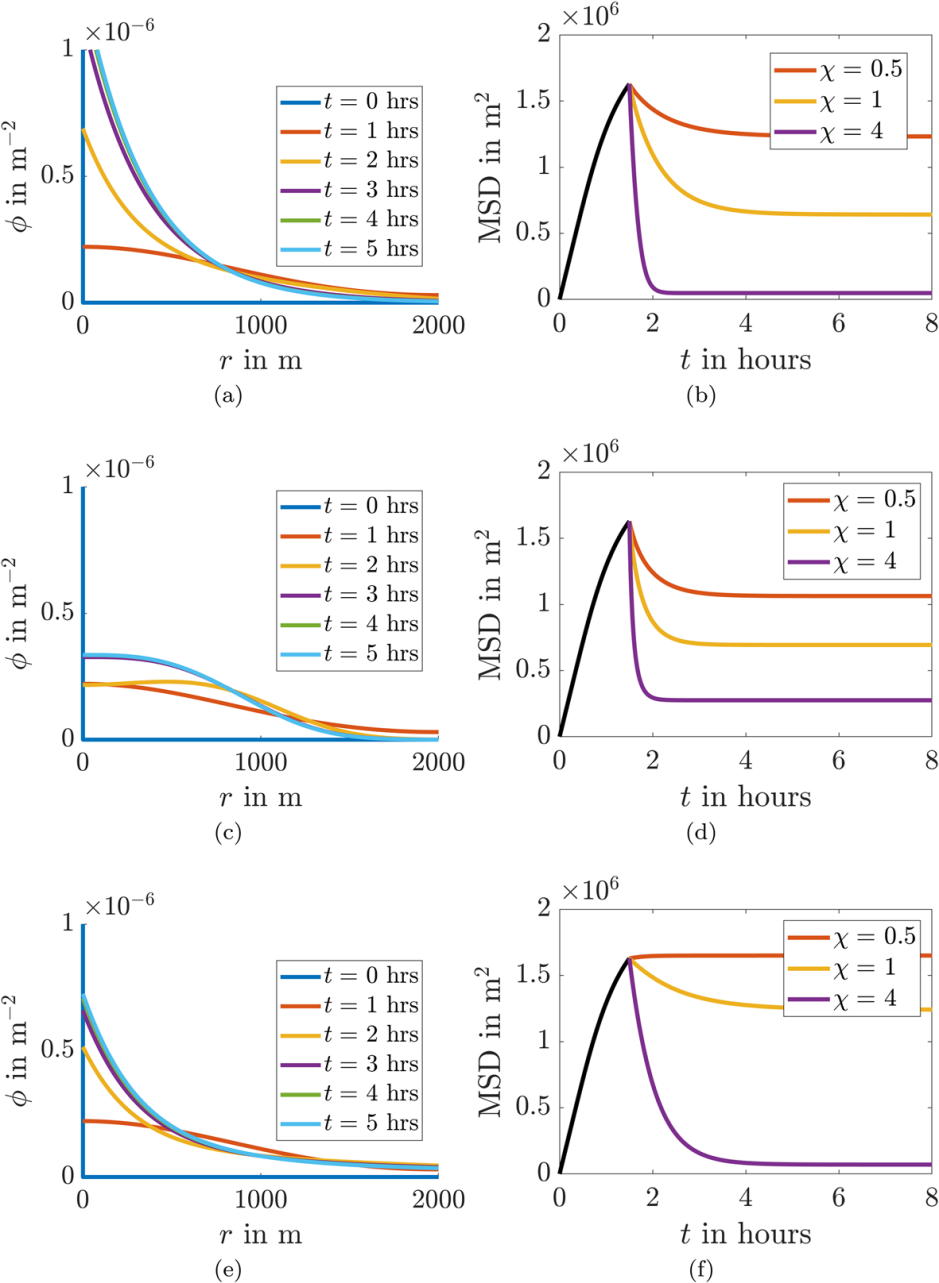
Population density simulations (left) and MSD plots (right) from Eq. (13) for different convection functions, $\varvec{v}$. All simulations occur on a circle of radius $R = 2000$m with $D=100$m^2^/s. In **a** and **b** $\varvec{v}=\chi \hat{\varvec{r}}$, **c** and **d** $\varvec{v}=\chi r^2 \hat{\varvec{r}}$ and **e** and **f** $\varvec{v}=\chi /(1+ r^2) \hat{\varvec{r}}$. In the left-hand figures $\chi =1$, whilst $\chi$ is specified in the legend of the right-hand figures (Color Figure Online)

We seek to test whether Eq. (13) can reproduce the MSD shown in phase 2 of Fig. 4a by considering forms of radial convection. In all cases we make $\varvec{v}$ piecewise non-zero. Namely, $\varvec{v}(t)=0$ for $t<1.5$hrs$=t_s$ because as we saw in Sect. 3.1 plain diffusion can account for the linear form of the increasing MSD in phase 1. For $t\ge 1.5$hrs$=t_s$ we set $\varvec{v} (t) =\chi f(r) \varvec{\hat{r}}$ and we consider three cases for the form of *f*, (i) uniform convection towards the origin, (ii) convection that increases with distance from the origin, and (iii) convection that decreases with distance from the origin. The cases of (i) and (ii) can be captured using $\varvec{v} (t) =\chi r^n \varvec{\hat{r}}$, with $n= 0$ being case (i) and $n>0$ being case (ii). Unfortunately, we cannot also consider $n<0$ as this produces a singularity in $\varvec{v}$ at the origin, thus, for case (iii) we use $\varvec{v} (t) =\chi /(1+r^n) \varvec{\hat{r}}$,

Figure 6 provides an overview of simulations with varying values of $\chi$ and *n*. In the left-hand column we observe the evolution of the probability density over time. In all cases we visualise the case where $\chi =1$. The right-hand column illustrates the extracted MSD. In all cases we observe that although the convection acts to draw the probability distribution towards the origin the shape of the MSD is concave and it tends to a non-zero steady state, where the diffusion spreading out $\phi$ balances the convection, which is driving the heterogeneity.

Although we could not have predicted the shape of the time-dependent MSD graphs without running the simulations we could have predicted that the simulations would have reached a non-zero steady state since the steady state form of Eq. (13) is actually solvable. Explicitly, at steady state Eq. (13) simplifies to

14 $$\begin{aligned} 0= \frac{\partial }{\partial r}\left( r\left( D\frac{\partial \phi _s}{\partial r}+\chi f(r) \phi _S \right) \right) , \end{aligned}$$

Using, the zero flux boundary conditions we can show that

15 $$\begin{aligned} \phi _s=\frac{\exp \left( -\int ^r_0\frac{\chi f(r_1) \text { d}r_1}{D}\right) }{2\pi \exp \left( -\int ^R_0\frac{\chi f(r)}{D} \text { d}r\right) }. \end{aligned}$$

and, thus, the steady state MSD is

16 $$\begin{aligned} \langle r_s^2 \rangle =\frac{1}{\exp \left( -\int ^R_0\frac{\chi f(r)}{D} \text { d}r\right) }\int ^R_0r_2^3\exp \left( -\int ^{r_2}_0\frac{\chi f(r)}{D} \text { d}r_1\right) \text { d}r_2. \end{aligned}$$

Since all terms in the integrands are positive $\langle r_s^2 \rangle$ must also be positive. We can observe from Eq. (16) that $\langle r_s^2 \rangle$ decreases as $\chi$ increases, or *D* decreases, thus, we can make the MSD head towards zero. However, this does not solve the problem that the MSD trajectories curves in the wrong direction.

Taking inspiration from the idea that altering *D* and $\chi$ could lead to a reduction of the MSD we now consider the possibility that the parameter values defining diffusion and convection are time dependent. We define

17 $$\begin{aligned} D(t)=D_c \left| 1- \frac{t}{t_c}\right| \text { and } \varvec{v}(t)=\chi _c t\varvec{\hat{r}}, \end{aligned}$$

where $D_c$, $t_c$ and $\chi _c$ are parameters to be fitted, with the restrictions that $D_c$ and $\chi _c>0$. The chosen temporal functional forms are linear in time for reasons of simplicity. We call Eq. (13) with functional terms defined by Eq. (17) the non-autonomous convection-diffusion model.

The chosen functional forms and interpretation of Eq. (17) are purposefully simple, but capture the expected dynamics. Initially, $\varvec{v}(t)\approx 0$ and, so, the model is diffusion dominated meaning that the bats spread out. However, as time increases *D*(*t*) decreases and $\varvec{v}(t)$ increases meaning that towards the end of the night the bats reduce their random motion characteristics and move in a more directed fashion towards the roost. We could produce much more complicated versions, but then this would require further justification. Moreover, we will see that the forms in Eq. (17) are enough to fit well against the data.

Having defined our model we can now fit the output MSD solutions to the data using nonlinear least-squares fitting algorithms built into MATLAB R2022b (Inc. 2022). Not only does the fitting algorithm provide point estimates for the best-fit parameter values, but it also supplies a 95% confidence interval for each value. The width of the interval indicates our uncertainty regarding the fitted values, with wider intervals correlating with more uncertainty. The values quoted here are presented to three significant figures, or two decimal places.

To provide a measure of ‘goodness-of-fit’ of the fitted curves we use the coefficient of determination. If the $\{y_i\}_{i=1}^N$ are the observations, $\{p_i\}_{i=1}^N$ are the predictions and $\langle y \rangle$ is the mean of the observations then the coefficient of determination is

18 $$\begin{aligned} {\mathcal {R}}^2=1-\frac{\sum _{n=1}^N(y_i-p_i)^2}{\sum _{n=1}^N(y_i-\langle y \rangle )^2}. \end{aligned}$$

${\mathcal {R}}^2$ measures the proportion of the variation in the MSD that is predicted by the time dependence and describes how well our model fits the observations, thus, the closer ${\mathcal {R}}^2$ is to 1 the better the fit.

**Fig. 7:**
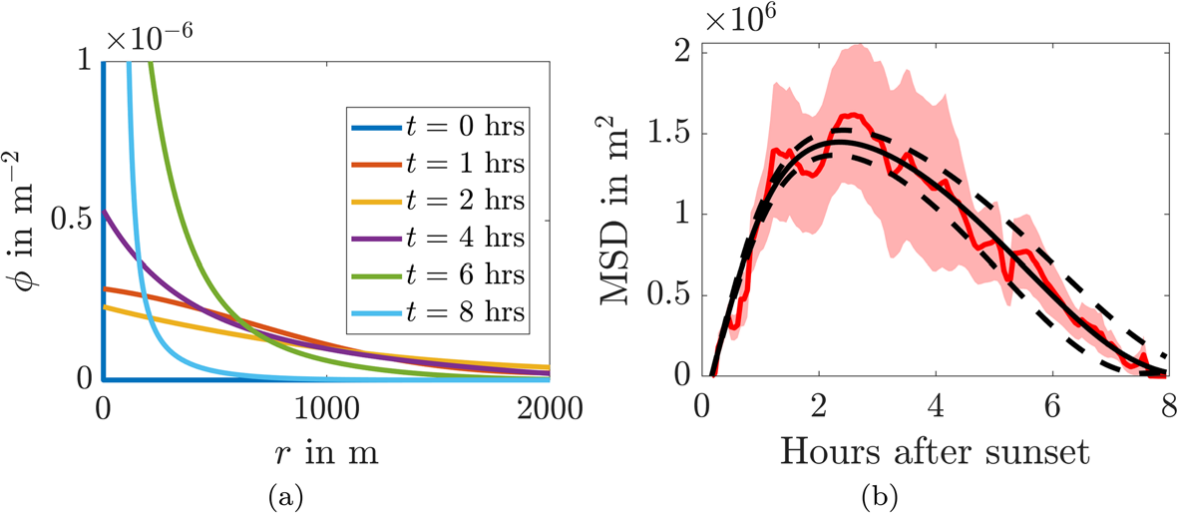
**a** Population density simulations and **b** MSD from Eq. (13) using temporally evolving diffusion and convection terms from Eq. (17) fitted to the data in Fig. 4a. The parameters and 95% confidence intervals are $D_c=100$m^2^/s, [94.4 106]m^2^/s; $t_c=8.23$h, [7.44, 9.00]h; and $\chi _c=0.83\times 10^{-5}$m/s^2^, [0.75, 0.91]$\times 10^{-5}$m/s^2^. In **b** The red line is the MSD trajectory and the ribbon represents the mean±standard error of the squared displacement trajectory data. The black solid line represents the simulated data with best-fit parameters. The dashed lines represent simulations using parameters from the upper and lower limits of the confidence intervals (Color figure online)

Upon fitting the non-autonomous convection-diffusion equation to the data shown in Fig. 4a we produce best-fit parameter values and 95% confidence intervals of $D_c=100$m^2^/s, [94.4 106]m^2^/s; $t_c=8.23$h, [7.44, 9.00]h; and $\chi _c=0.83\times 10^{-5}$m/s^2^, [0.75, 0.91]$\times 10^{-5}\,$m/s^2^. The density of the bat population is shown in Fig. 7a, whilst the fitted MSD curve, which has ${\mathcal {R}}^2=0.96$, can be seen in Fig. 7b. We observe that the non-autonomous convection-diffusion equation fits the data extremely well, but the density profiles in Fig. 7a do not fit with the expected behaviour as suggested in the ecology literature.

As time tends towards dawn the strength of the convection term increases causing the bat population density to move towards the roost. However, the convection term causes the bats nearest to the roost to return first. This is currently against the narrative in bat ecology that says that bats stay out as long as possible (Pyke 1984) and only return to the roost just before dawn to maximise their foraging potential (McAney and Fairley 1988; Murray and Kurta 2004). In the next section we define a new mechanism that accounts for bats returning to the roost due to the bats furthest out returning first, which would fit better with the current knowledge of bat behaviour.

#### Leap Frogging

**Fig. 8:**
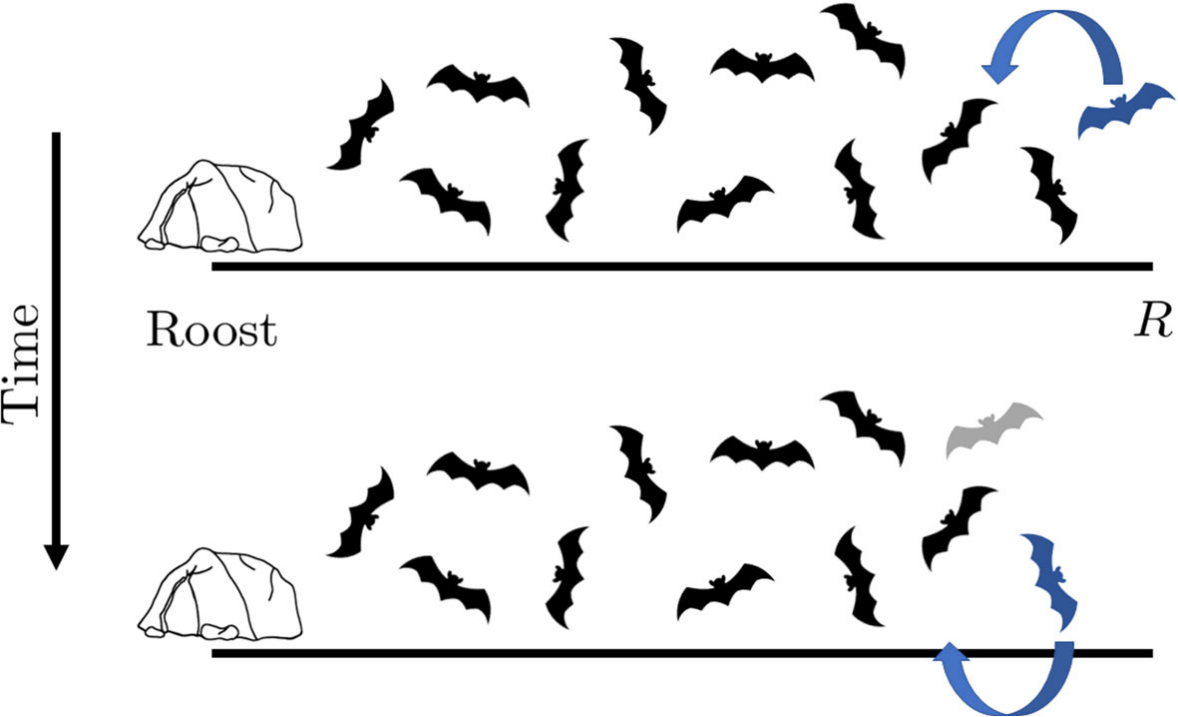
Schematic diagram illustrating the leap frogging strategy of movement in one-dimension. The black bats are all moving randomly. The furthest out, highlighted in blue convects until it is no longer the furthest out. Its motion then becomes diffusive. The bat that is now furthest out starts to convect towards the roost and the process repeats (Color figure online)

Instead of pulling the bats towards the roost in this section we generate a motion mechanism pushes the bats on the periphery back to the roost. We term this form of motion ‘leap frogging’ because it mirrors the idea that bats on the periphery will choose to fly towards the roost until they are no longer the furthest bat away from the roost. Once the convecting bat is no longer the furthest out from the roost it stops convecting and returns to moving randomly. The new bat that is furthest out starts to convect towards the roost and the process begins again. Note that the furthest out bat becomes the edge of the domain and no randomly moving bat is able to move past it. Over time this form of motion will cause the bat population to tend towards the roost, as shown in Fig. 8.

Of course, with this strategy comes the questions: (i) why would the furthest out bat be the only one convecting? And (ii) how would the furthest out bat know that they are the furthest one out?

We answer (i) by appealing to a number of different aspects linked to self-preservation. Firstly, a bat on the periphery is more vulnerable to predators as it is not surrounded by other bats that would act as potential targets that could take the predator’s attention. Secondly, the furthest out bats have furthest to travel back to the roost, so it is likely that they would be the ones to start returning first. Equally, bats closer to the roost have less far to travel and can continue to forage for longer. Thirdly, if the rest of the bat population has found suitable foraging areas closer to the roost the furthest out bat is wasting energy by flying further.

To answer (ii) we focus on the recent research into bat calls that suggests that there is a lot of contextual information in their calls beyond just use in echo location (Prat et al. 2016; Genzel et al. 2019). Not only do bats gain a lot of spatial information from hearing other bats calling they will also be able to discern that they are the furthest out if they are unable to hear calls emanating from all directions. So, if a bat can hear calls from all surrounding directions it can infer that it is not the furthest out and, thus, free to forage randomly. However, as soon they become the furthest out bat the calls will no longer surround them, triggering a change in their behaviour.

The stochastic form of this motion can be seen considered in Henley (2022). However, using stochastic-to-deterministic scaling arguments (Woolley et al. 2011c; Woolley 2011) we have found that the motion can be interpreted and included in the presented framework of diffusion-convection equations. Further, using the deterministic form produces tractable equations, which can be analytically approximated. From these approximations we can fit the temporally evolving convection term to provide a MSD that matches the data. Although, this can also be done stochastically, using Approximate Bayesian Computation (Blum 2010; Prangle 2015; Henley 2022), this would require many thousands of stochastic simulations to fully explore the parameter regime. Because of these reasons we move forward using deterministic equations only.

The key observation is that convecting the boundary bats towards the roost is the same as casting the diffusion motion on an apically shrinking domain, *i.e.* the domain shrinks only at the edge. The derivation of reaction-diffusion equations on domains undergoing growth or shrinkage is standard, and involves considering an elemental volume that moves with the flow due to domain evolution. By applying Reynolds’ transport theorem (Acheson 1990) in addition to the standard diffusive movements terms we generate an additional advective term which accounts for the transport of material around the domain, as well as a source/sink term that accounts for concentration/dilution which arises due to volume changes. We skip further specifics of the derivation for brevity, but note that further details can be found in any of the following sources (Barrass et al. 2006; Crampin 2000; Crampin and Maini 2001a, b; Crampin et al. 1999, 2002a, b; Madzvamuse and Maini 2007; Neville et al. 2006) and the simulation codes accompanying this paper can be found at https://github.com/ThomasEWoolley/Bat_motion.

In short, if we assume that the diffusion is occurring on an origin-centred disk with time dependent radius *R*(*t*), we can use a Lagrangian description of the coordinates to map the motion onto a stationary domain, such that the diffusion equation becomes a convection-diffusion equation with time-dependent coordinates,

19 $$\begin{aligned} \frac{\partial \phi }{\partial t}=\frac{1}{R(t)}\nabla _{\varvec{\rho }}^2 \phi +\frac{\dot{R}}{R}\varvec{\rho }\cdot \nabla _{\varvec{\rho }} \phi , \end{aligned}$$

where the position vector and derivatives are all with respect to the stationary coordinate $\varvec{\rho }=(X,Y)$ and are linked to the time dependent coordinates $\varvec{r}=(x(t),y(t))$ through the uniform scaling $\varvec{r}=R(t)\varvec{\rho }$. The equation once again has zero-flux boundary conditions and an initial delta function of concentration at the origin.

We observe that we are still going to be simulating a convection-diffusion equation, however, Eq. (13) fundamentally assumes that the domain is stationary and the convection term arises as a temporally uniform, but spatially homogeneous driving force pushing the bats back to the roost. Alternatively, the convection in Eq. (19) arises due to the temporally shrinking domain driving the bats back to their roost.

Next, we will discuss the form of the shrinking rate. We make the assumption that the diffusion rate is larger than the rate at which the domain changes size. In this case, the solution should approximate the homogeneous steady state on a bounded domain to a good approximation. We offer some verbal reasoning as to why this assumption, although not exactly correct, should be valid on both short and long time scales.

Over short time scales any heterogeneity will be constrained to a thin region near the edge, thus, the homogeneous solution will hold true over most of the domain. Over long time scales any gradients that are generated will be spread out over physical distances of kilometres. In addition to this justification we will demonstrate a posteriori that this assumption does produce the desired behaviour even though it may be violated in reality towards the end of the night.

Using the assumption that our solution is near a uniform steady state Eq. (11) says that $\phi =1/\left( \pi R(t)^2\right)$. Moreover, Eq. (12) tells us that $\langle r^2\rangle = R(t)^2/2$. From this, we can choose the radius so that the expected MSD matches the observed MSD from the radio-tracking data.

In the next section *R*(*t*) will be fit to the data exactly using a nonlinear least-squares fit. However, in this section we demonstrate that our assumptions work by choosing a form of the domain radius, *R*(*t*), to give a convex MSD. To match the two phases of the observed motion we use a piecewise form of *R*(*t*), such that $R(t)=R_0$ for $t<1.5$hrs and, so, the domain has a constant radius during phase 1. Then in phase 2 $t>1.5$hrs, we choose $R(t)^2=R_0^2(1-\alpha (t-1.5)^2)$, so that the domain radius shrinks like a negative parabola, which decreases from the initial value of $R_0$. Further, to fix $\alpha$ we assume that the MSD reaches zero at $t=8$hrs, *i.e.* $\alpha =1/(8-1.5)^2$.

**Fig. 9:**
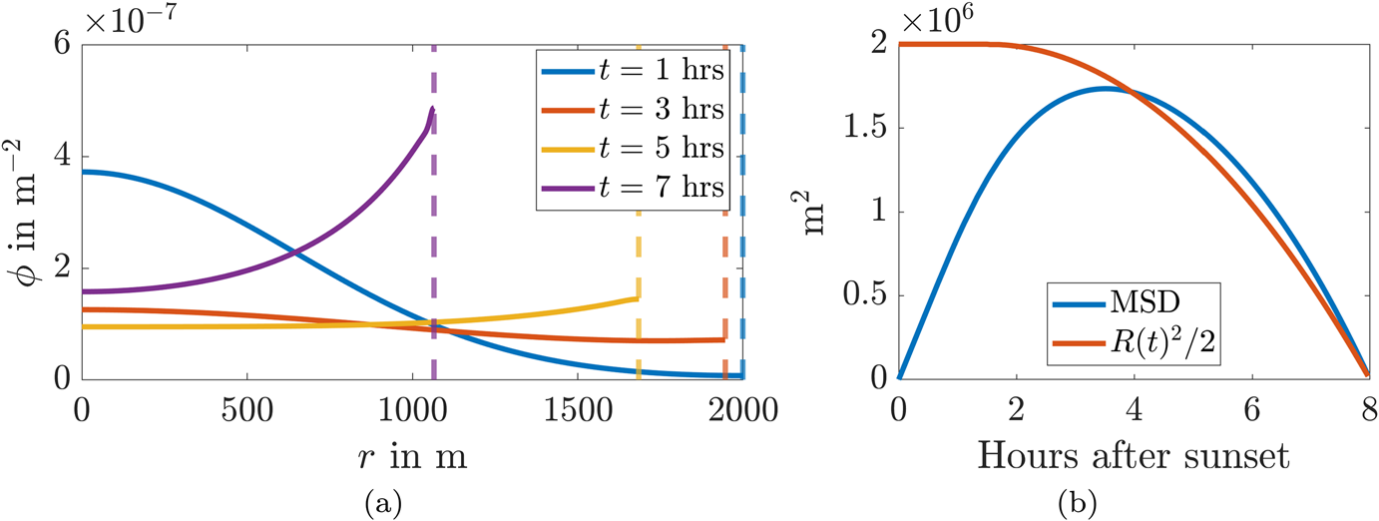
**a** Results of a diffusion simulation on a shrinking domain at different times given in the legend. The dashed line presents the boundary location at the given times. **b** The extracted values of *MSD*, with $R(t)^2/2$ for comparison. The initial condition is a delta function at $r = 0$ and the time-dependent radius of the domain is $R(t)=R_0$ for $t<t_s$ and $R(t)=R_0\sqrt{1- \left( (t-t_s)/(8-t_s)\right) ^2}$, with $R_0 = 2000$m, $t_s = 1.5$ hours and $D = 100\,$m^2^/s (Color Figure Online)

The probability density and MSD of $\phi$ undergoing diffusion on a shrinking domain with time-dependent radius, *R*(*t*), is shown in Fig. 9. During phase one the domain’s width is constant and we observe that the probability density, shown in Fig. 9a, spreads across the domain due to the diffusion process. This corresponds with the MSD increasing approximately linearly in Fig. 9b for $t<1.5$hrs.

For $t>1.5$hrs the domain starts to shrink, as defined above, and we see that the MSD decreases, producing a convex shape. In Fig. 9b we also plot $R(t)^2/2$ the theoretical MSD that would occur if $\phi$ was homogeneous across the domain. We see that the MSD and $R(t)^2/2$ well approximate each other for $t>3$hrs. We can understand this by considering the approximate homogeneity of $\phi$ in Fig. 9a, where we observe that $\phi$ has a very shallow gradient across the domain for $3<t<7$hrs. Even when $t=7$ and $\phi$ doubles over the domain this doubling occurs over 1 km, thus, the gradient would be very small over this distance. Finally, during the last hour, when the homogeneity of $\phi$ would start to be violated the MSD is small enough that any errors simply get smaller and the approximation actually gets better (compare the two curves in Fig. 9b at $t\approx 8$hrs).

As a result, we conclude that, even when our assumption does not hold true, and the probability density is not uniform, the simulation produces the desired behaviour. Using the close approximation of the MSD and $R(t)^2/2$, we can fit *R*(*t*) to the radio-tracking data, which will do in the next section.

#### Data Fitting

**Fig. 10:**
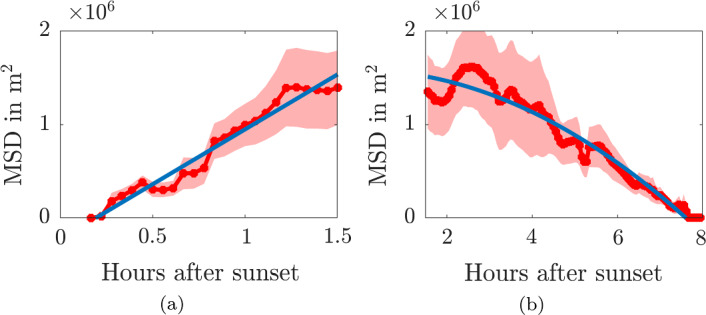
Fitting the two phases of bat MSD data. **a** Phase 1 presents the data for $t<1.5$hrs after sunset. The data is fitted with a straight line. **b** Phase 2 presents the data for $t>1.5$hrs after sunset. The data is fitted with a quadratic polynomial. The ribbons represent the standard error

Figure 10 presents the data from Sect. 2 separated in the two phases of before 1.5hrs after sunset and after 1.5hrs after sunset. The data from phase 1 is fitted with a straight line, $MSD=\alpha _{11}t+\alpha _{10}$, (Fig. 10a) and the data from phase 2 is fitted with a quadratic polynomial, $MSD=\alpha _{22}t^2+\alpha _{21}t+\alpha _{20}$, (Fig. 10b). The polynomial regression was, once again, obtained using least-squares algorithms built into MATLAB R2022b (Inc. 2022).

From the fitted values and the identification that $4D=\alpha _{11}$ we can calculate that the diffusion rate of the bats is $D=81.7$m^2^/s. This means that every second a population of bats will spread out into an area of approximately 82 m^2^. To convert this to a speed we need to use a standard time scale over which the bats move. We consider the time scale to be on the order of 1 s (or higher). To generate a bat speed, $v=r(t)/t$, we note that the diffusion rate can be linked to the area that the bats spread over, $\pi r(t)^2$, in the given time scale, *i.e.* $D=\pi r(t)^2/t$. Manipulating these equations we find that

20 $$\begin{aligned} v=\sqrt{\frac{D}{\pi t}}. \end{aligned}$$

Substituting the fitted values and the time scale into Eq. (20) we generate a speed of $v=5.10 \pm 0.11$m/s. Further, as we see in Fig. 10, there is a delay between sunset and the MSD observably increasing. From the linear fit we can calculate the delay as $t_d=-\alpha _{10}/\alpha _{11}=698$s, or approximately 11.6 min.

For phase 1 the linear fit to the data has a goodness of fit of ${\mathcal {R}}^2=0.96$. The quadratic fit of phase 2 has ${\mathcal {R}}^2=0.95$.

By fitting these two phases separately we can combine them directly. Thus we propose to apply a diffusion model of movement acting on a shrinking domain to both phases. The diffusion rate is $D=81.7$m^2^/s (taken from phase 1) and the domain size *R*(*t*) (taken from phase 2) is linked to the fitted MSD curve through $MSD=R(t)^2/2$, explicitly,

21 $$\begin{aligned} R(t)=\sqrt{2\left( \alpha _{22}t^2+\alpha _{21}t+\alpha _{20}\right) }. \end{aligned}$$

From this definition we can extract an estimate for the CSZ as $R(0)=\sqrt{2\alpha _{20}}=1780$m which is the largest domain that the bats can spread out into since *R*(*t*) decreases as *t* increases.

**Fig. 11:**
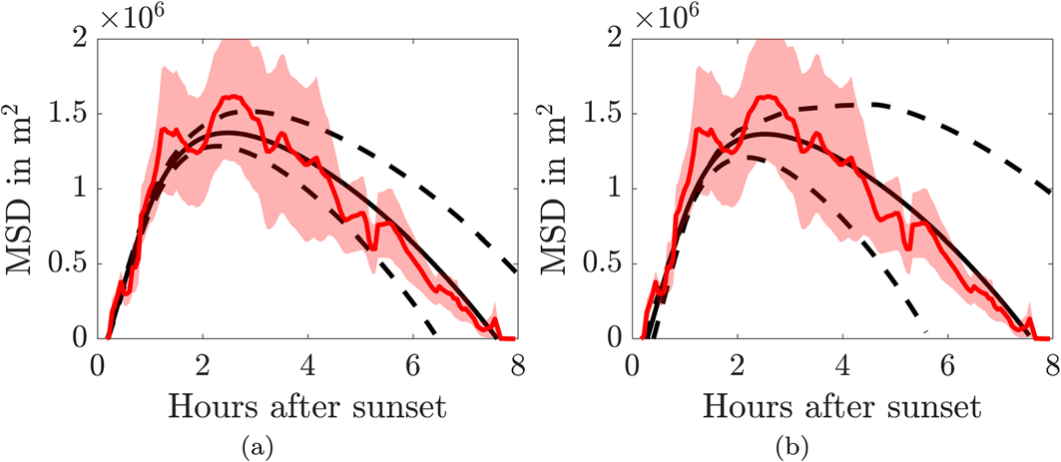
MSD of diffusing agents on a shrinking domain. The red line is the MSD trajectory and the ribbon represents the mean±standard error of the sqaured displacement trajectory data. The black lines represent simulated data. The diffusion cofficient is $D=a_{11}/4$ and the radius of the domain, *R*(*t*), is given by Eq. (21). The solid line uses the best-fit parameter values, whilst the upper and lower dashed lines use the upper and lower values from the 95% parameter confidence intervals. The parameters are specified in Table 1. **a** The parameters are fitted with $t_s=1.5$ hours. **b** The parameters are refitted with $t_s$ included in the fitting (Color figure online)

Figure 11a shows the two phases simulated together under the above extracted parameter values and functional forms. The goodness-of-fit value of the black line, which uses the best-fit parameters is ${\mathcal {R}}^2=0.93$. The best-fit parameters are specified on the left of Table 1.

By using Eq. (21) we no longer have to consider a piecewise form of *R*(*t*) that is constant for $t<1.5$hrs and decreasing for $t>1.5$hrs. Equation (21) is able to account for both phases simultaneously. Equally, the value of *D* is constant throughout the simulation.

Up until now we have fixed the transition time between dispersion and roost return at $t_s=1.5$ hours. We now extend our method to be piecewise linear and quadratic, such that a linear curve is fitted for $0<t\le t_s$ and a quadratic is fitted for $t_s<t$. Upon fitting the piecewise curve to the data we generate the best-fit parameter and confidence intervals as presented in the right of Table 1. We observe that the best-fit parameter for $t_s=1.46$ hours, which confirms the ecologists anecdotal knowledge that the first 90 min after sunset is a good prediction for the major foraging period. Combining these piecewise parameters into the single simulation we generate the best-fit curve illustrated in Fig. 11b that is practically indiscernible from that shown in Fig. 11a, however, the error bounds provided by the upper and lower curves are wider.

**Table 1:** Best-fit parameters and 95% confidence intervals for the data fitted with a linear-quadratic piecewise function, where a linear fit occurs for $0<t\le t_s$ and a quadratic fit occurs for $t_s<t$

Parameter	Unit		Best-fit value	Confidence interval		Best-fit value	Confidence interval
$a_{11}$	m^2^/s		327	[294,360]		335	[301,370]
$a_{10}$	m^2^		$-$2.28$\times 10^5$	[$-$3.34, $-$1.22]$\times 10^5$		$-$2.46$\times 10^5$	[$-$3.54, $-$1.38]$\times 10^5$
$t_s$	hours		1.5	Fixed		1.46	[0.60, 2.32]
$a_{22}$	m^2^/s^2^		$-$2.04$\times 10^{-3}$	[$-$2.56,$-$1.52]$\times 10^{-3}$		$-$2.11$\times 10^{-3}$	[$-$2.62,$-$1.60]$\times 10^{-3}$
$a_{21}$	m^2^/s		$-$2.13	[$-$20.1, 15.8]		0.48	[$-$17.3, 18.2]
$a_{20}$	m^2^		1.58$\times 10^{6}$	[1.45, 1.73]$\times 10^{6}$		1.57$\times 10^{6}$	[1.43, 1.70]$\times 10^{6}$

The parameters on the left assume that $t_s=1.5$ hours is fixed, whilst the parameters on the right include $t_s$ in the fitting process

As noted in Fig. 4b increasing the track interpolation interval from $\Delta t=200$s will lead to variations in the best-fit parameter values stated here. However, since the overall shape of the curve does not change greatly our framework would still fit the data and the variability of the numerical values would be no worse than the ecological data itself. Should the interested reader want to vary $\Delta t$ all of the data and accompanying MATLAB codes can be found at https://github.com/ThomasEWoolley/Bat_motion.

### Comparing Non-autonomous Convection–Diffusion and Leap Frogging Mechanisms

Figures 7b and 11 demonstrate that both the non-autonomous convection-diffusion model and the leap frogging mechanism are able to fit the observed data well. Thus, we need a metric to distinguish between the two motion descriptions. We focus on using the information provided by the density plots in Figs. 7a and 9a, where we see that a major difference between the two mechanisms is the placement of the density peak. In the case of the non-autonomous convection-diffusion model the peak appears close to the roost (the bats are being attracted, or pulled towards the roost), whereas in the leap frogging model the density peaks at the boundary (the bats on the boundary are being pushed towards the roost).

**Fig. 12:**
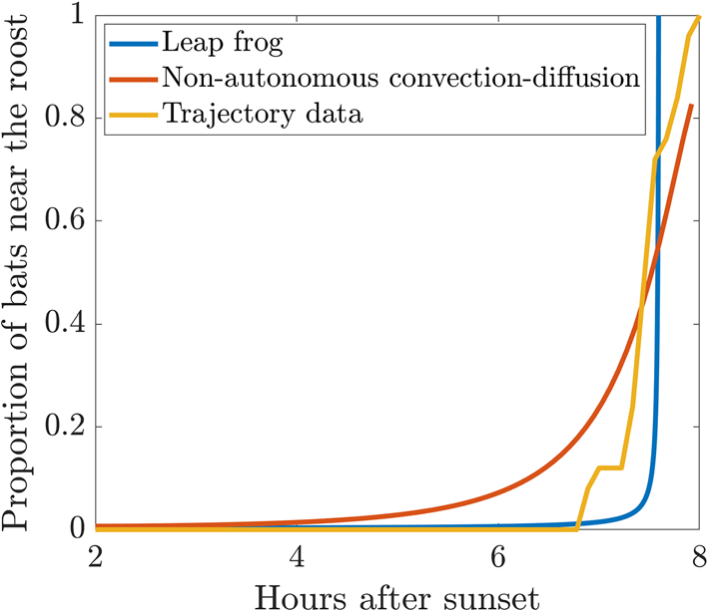
Comparing the density of returning bats within 100 m of the roost across the two simulated mechanisms of non-autonomous convection-diffusion and leap frogging with the trajectory data (Color Figure Online)

As mentioned at the end of Sect. 3.2.1 the current narrative of bat behaviour suggests that bats would prefer to maximise their foraging time and stay out as long as possible (McAney and Fairley 1988; Murray and Kurta 2004), which would align closer with the idea of the leap frogging mechanism because in this case it is the bats that are furthest out that are returning first, whilst those nearer to the roost continue to forage (Speakman 1991). We test this idea in Fig. 12 by calculating the proportion of bats that are within 100 m of the roost through integrating the density profiles of the two mechanisms and seeing how they change towards the end of the night. We note that 100 m is an arbitrary distance from the roost to use, but offers a clear difference between the two mechanisms we are trying to decide between. We then compare these simulated curves with data as we extract the time at which each bat is detected within 100 m and is not detected beyond 100 m thereafter. Since we know that all the bats returned to the roost we assume that the bat never travels beyond 100 m again. From extracting this time we can generate an approximate density of returning bats throughout the night.

Figure 12 illustrates our central idea that under the non-autonomous convection-diffusion model bats return earlier than under the leap frog model. Moreover, the non-autonomous convection-diffusion model predicts that not all bats ($\approx 20\%$) will return, even by dawn, which is problematic as it is known that bats are extremely sensitive to light (Rossiter et al. 2000). In comparison, the data line lies in between the two proposed mechanisms, suggesting that it may be a combination of both processes that defines the bat behaviour; a pull from the roost to head home and a push from bats returning from the furthest out parts of the CSZ. However, the rapid increase towards the end is much more in line with the ecological theory that the bats tend to stay out as long as possible before returning home (Pyke 1984).

We note that the assumption that the bats never travel beyond 100 m again between the detected time and dawn is a weakness of the data comparison. The irregular intervals between the detections means that it is possible that the bats did fly outside the radius of 100 m. However, this would simply cause the final return time to be later making the data fit better with the curve from the leap frog simulation. A better source of data would be to place a static microphone detector close to the roost. This is what was done in (McAney and Fairley 1988), where it can be observed that the number of detections rapidly increases within the last hour, which is consistent with our results. Thus, although more data needs to be collected to really test our two hypotheses, the leap frog motion description appears to be more consistent with the observed data and bat behaviour hypothesis.

## Discussion

In this paper we have considered the mean squared displacement of a foraging bat population extracted from survey data that occurred in Devon during May-June of 2016. We observed that there was a clear separation of motion types during the night, an initial dispersal phase and a slow drift back to the roost. We used partial differential equations to model the motion and we have discovered two mechanisms that fit the data, one pulls the bats towards the roost (non-autonomous convection-diffusion), whilst the other pushes the bats from the boundary (leap frogging).

Having discovered two mechanisms that fit the data, we must consider the ability of testing the mechanisms and deciding between them. In Sect. 3.3 we saw that the major difference between the non-autonomous convection-diffusion model and the leap-frog model is that the local maximum are at opposite ends of the bat density profile. In the non-autonomous convection-diffusion model the density peaks at the roost, whilst in the leap frog model the density is higher on the boundary. Comparing the mechanisms to data and ecologist narrative we suggest that either a mix of the mechanisms, or the leap frogging model are the best fit. Not only do bats want to stay out from the roost as long as possible to maximise foraging, suggesting that bats on the outside moving in first is more likely, but also the non-autonomous convection-diffusion model has a problem in that it predicts that there will be a non-zero population not in the roost at sunrise. We suggest further testing of our hypotheses with static detector microphone arrays that can be placed out in the field and these can be used to track the call density throughout the night. Such experiments are less labour intensive than bat tracking and provide constant surveillance through the night, thus, we can generate much more data. Having this data would immediately allow us to see when and where the density of returning bats tends to concentrate, *i.e.* at the roost, or move in from the boundary.

Considering the two phases observed in the data, the initial rapid dispersal from the roost in phase 1 can be explained by competition for resources. If the colony spreads out across the domain, each individual has a larger area to themselves, and therefore more resources available to them.

We have shown that a diffusion model provides a good description of motion in phase 1 as it produces a linear growth in the MSD. From the two mechansims derived in this paper we have generated a range of possible diffusion rates, $D=73.5$-106m^2^/s, which can be interpreted as an approximate straight line speed of $v=4.84$-5.81m/s. This fits well with data as, during foraging, greater horseshoe bats have been recorded flying at speeds of 8.1m/s (Duvergé and Jones 1994). Thus, we can be reasonably confident in the application of this model to describe the motion of the bats at least initially.

However, we must consider the limitations of our descriptions of bat motion. The bat roosts we consider here are in a rural area, thus bats are able to spread out in all directions. In urbanised areas bat flight directions will be more impeded since bats are light averse, and show avoidance of road noise (Finch et al. 2020b, c; Barlow et al. 2015). Hence, if we were to repeat this study in a different location we would have to revisit the assumption that bat motion was equally likely in all directions. Further, many bat species (such as greater horseshoe bats) prefer to travel along linear features such as hedgerows and rivers where insects are abundant and navigation is easy (Barlow et al. 2015), suggesting that the movement may be better described as one-dimensional, rather than two-dimensional. Even if we were to reduce the dimension of the model and only consider bat motion in one-dimension, the MSD of diffusion is still linear with respect to time. Although the gradient would be 2*D*, rather than 4*D*, which would cause us to record a doubled straight line speed (9.68–11.62 m/s) which would still be within a bat’s plausible speed range (Woolley et al. 2017).

Critically, it does not matter whether the motion is predominantly one-dimensional (in that the bats prefer to follow linear geographical features *e.g.* greater horseshoe bats), two-dimensional (in that the bats will spread out and fill open spaces, *e.g.* noctule bats), or a mixture of the two; the diffusion model will still be able to match the data (up to a factor of 2), which is why the MSD is a useful description of motion. This means that, regardless of the specific flight features, our derived ranges of bat dispersion rates will be of the right ‘order of magnitude’, which is often as accurate as we can be in ecological settings due to the variability in bat species, geography and climate.

We must also consider the fact that bat motion is actually three-dimensional and we are missing height data. This lack of altitude information is a fundamental limitation of the current technology and tracking practices and we do know that it limits our understanding of bat behaviour as it has been shown that some species produce different call types at different altitudes (Jensen and Miller 1999). Our only hope is that future developments in technological miniaturisation will lead to detectors that can be attached to bats and provide full three-dimensional tracking. If full three-dimensional data becomes available the model presented here can be easily updated to include the third dimension through appealing to cylindrical polar coordinates.

One possible interpretation of the leap frog model is that the bats depend on the calls around them to help navigate their environment. It is known that a common foraging strategy amongst some bat species is to eavesdrop on the hunting calls of other bats to easily and quickly locate hunting grounds (Roeleke et al. 2020; Egert-Berg et al. 2018). This strategy is most common in landscapes dominated by cropland, where prey is difficult to find for a single bat due to patchy or unpredictable insect distribution and is uncommon in woodland where insect distribution is more reliable. Eavesdropping allows bats to locate areas with insects by following bats that have already found these hunting grounds.

It is likely that the density of bats far away from the roost is often low enough that they do not hear calls from other bats constantly. However, foraging bats are not stationary, instead they fly around searching for prey (Jones and Rayner 1989). We suggest that the mechanism sending bats towards the roost could be the absence of calls close to them: if a bat spends a significant period of time in silence, without hearing calls from other bats, or if all the calls come from one direction, the bat would decide to head closer to the roost, towards where it will find other members of the colony.

This mechanism would suggest that during the returning phase of the night bats could be made to return slower and forage longer if they thought that there were bats beyond their location. An acoustic playback experiment could be designed to test this hypothesis as we would be able to asses whether microphones playing foraging calls at the edge of a CSZ would lead to a slower return. However, in such an experiment ethical decisions would need to be made regarding the bat population’s safety, since encouraging bats to stay out from their roost for longer may lead them to be more vulnerable.

The quadratic form of the radius of the shrinking domain, given by Eq. (21), suggests that bats move very slowly towards the roost at the start of phase 2, and the rate of return increases as time goes on. We consider two possible explanations for this. Firstly, a bat located far from the roost is likely to have less knowledge of its surroundings, and therefore may be more inclined to employ the eavesdropping strategy as it is more reliant on the behaviour of other bats in the colony. As the bat travels closer to the roost, navigation becomes easier because the landscape is more familiar, and the bat is therefore able to return directly to the roost rather than relying on others.

We note that for the species we are considering (greater horseshoe bats) it is not clear whether eavesdropping is used as a foraging strategy, but regardless, it is clear that they can recognise conspecifics based on echolocation calls (Barclay and Jacobs 2022), while other horseshoe species can even use acoustic information to determine the sex of individuals (Schuchmann et al. 2012). Therefore, it is reasonable to hypothesise that bats use the absence of echolocation calls, perhaps experienced over an extended time period, as a cue for returning towards a roost.

Alternatively, the time of night may provide behavioural cues. Early in the night, the bat knows that there are several hours before the sun rises and it can therefore continue to forage safely and eavesdrop on other bats to locate abundant hunting grounds. Later on in the night, the sense of urgency to return to the roost becomes more pronounced, as a bat does not want to be stranded far from the roost, without shelter, when the sun rises, and therefore return travel becomes more urgent as the night progresses to dawn. Moreover, it is possible that bats do not want to be further from the roost than the rest of the colony once they have eaten, and therefore start to travel back after foraging has succeeded.

Of course our conclusions regarding bat motion are not the whole story. Bats are not restricted to returning to the same roost each night, indeed many of the trajectories in the initial data recorded bats starting in one roost and heading towards another. So, there is work to be done extending these results to include multiple roost locations. Equally, this work is only applicable to greater horseshoe bats during the survey months because it is known that different bat species fly in different manners and prefer different habitats, whilst the same species can present different flight patterns during different times of the year (Voigt et al. 2017b). Thus, although we have provided the first steps to understanding bat motion characteristics there are more questions to be answered.
